# Snake mitochondrial genomes: phylogenetic relationships and implications of extended taxon sampling for interpretations of mitogenomic evolution

**DOI:** 10.1186/1471-2164-11-14

**Published:** 2010-01-07

**Authors:** Desirée A Douglas, David J Gower

**Affiliations:** 1 Division of Evolutionary Molecular Systematics, Department of Cell and Organism Biology, University of Lund, Sölvegatan 29, 22362 Lund, Sweden; 2 Department of Zoology, The Natural History Museum, Cromwell Road, London, SW7 5BD, UK

## Abstract

**Background:**

Snake mitochondrial genomes are of great interest in understanding mitogenomic evolution because of gene duplications and rearrangements and the fast evolutionary rate of their genes compared to other vertebrates. Mitochondrial gene sequences have also played an important role in attempts to resolve the contentious phylogenetic relationships of especially the early divergences among alethinophidian snakes. Two recent innovative studies found dramatic gene- and branch-specific relative acceleration in snake protein-coding gene evolution, particularly along internal branches leading to Serpentes and Alethinophidia. It has been hypothesized that some of these rate shifts are temporally (and possibly causally) associated with control region duplication and/or major changes in ecology and anatomy.

**Results:**

The near-complete mitochondrial (mt) genomes of three henophidian snakes were sequenced: *Anilius scytale*, *Rhinophis philippinus*, and *Charina trivirgata*. All three genomes share a duplicated control region and translocated tRNA^LEU^, derived features found in all alethinophidian snakes studied to date. The new sequence data were aligned with mt genome data for 21 other species of snakes and used in phylogenetic analyses. Phylogenetic results agreed with many other studies in recovering several robust clades, including Colubroidea, Caenophidia, and Cylindrophiidae+Uropeltidae. Nodes within Henophidia that have been difficult to resolve robustly in previous analyses remained uncompellingly resolved here. Comparisons of relative rates of evolution of rRNA vs. protein-coding genes were conducted by estimating branch lengths across the tree. Our expanded sampling revealed dramatic acceleration along the branch leading to Typhlopidae, particularly long rRNA terminal branches within Scolecophidia, and that most of the dramatic acceleration in protein-coding gene rate along Serpentes and Alethinophidia branches occurred before *Anilius *diverged from other alethinophidians.

**Conclusions:**

Mitochondrial gene sequence data alone may not be able to robustly resolve basal divergences among alethinophidian snakes. Taxon sampling plays an important role in identifying mitogenomic evolutionary events within snakes, and in testing hypotheses explaining their origin. Dramatic rate shifts in mitogenomic evolution occur within Scolecophidia as well as Alethinophidia, thus falsifying the hypothesis that these shifts in snakes are associated exclusively with evolution of a non-burrowing lifestyle, macrostomatan feeding ecology and/or duplication of the control region, both restricted to alethinophidians among living snakes.

## Background

**Figure 1:**
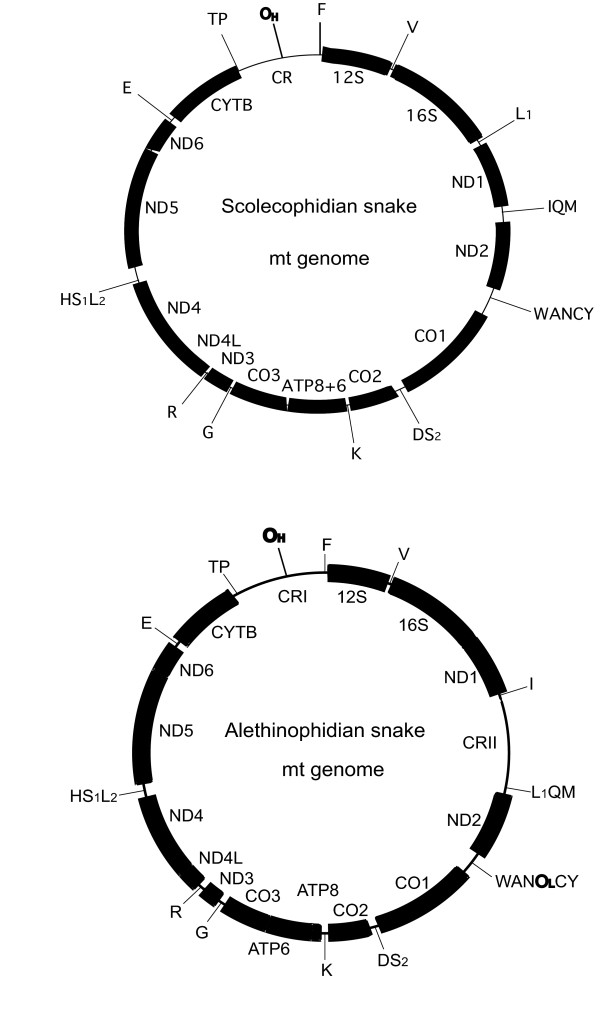
**Gene order of scolecophidian and alethinophidian mt genomes**. The scolecophidian mt genome resembles the standard vertebrate mt genome except for the lack of O_L_. Note also that in *Leptotyphlops humilis *tRNA^GLN ^is translocated. The alethinophidian mt genome has a duplicate control region and translocated tRNA^LEU^. The O_L _is present.

Vertebrate mitochondrial (mt) genomes have been the subject of many studies of phylogeny and evolutionary genetics and genomics, by virtue of characteristics such as their manageable size and generally conserved gene content and order. Interest in snake mitogenomics has focused on topics as diverse as gene duplications, truncations and order rearrangements [1-4] Fig. 1], attempts to resolve the contentious phylogenetic relationships of the major snake lineages [5] and references therein] and, in addition, inferring patterns and understanding processes of genome functionality and metabolic protein evolution [3,6].

The most basal split within extant snakes is between Scolecophidia (blind- and wormsnakes) and Alethinophidia. There is a large asymmetry in the number of extant species in these two clades with only 15% of species belonging to Scolecophidia. Within Alethinophidia, most species (c. 2,500) belong to the 'advanced' snake clade Caenophidia, while the remaining (c. 180) species comprise the paraphyletic Henophidia, whose phylogenetic intrarelationships are contentious [7].

Complete or near-complete mt genome sequences have been published for over 20 snake species [1-6,8-10]. Two innovative and important recent studies [3,6] inferred phylogenetic relationships among snakes based on complete mt genome sequence data for 10 and 11 species (and genera) respectively, and used this framework to examine mt genome evolution. These two studies demonstrated that snakes are unusual among vertebrates in their accelerated mt gene evolution, in that protein-coding gene branch lengths along certain branches (especially those 'leading' to all snakes and to Alethinophidia) are disproportionately long relative to rRNA genes, and that episodic bursts of gene- and branch-specific evolution underpinned adaptive remodeling of metabolic proteins early in snake evolution that are potentially causally associated with control region duplication and/or major changes in anatomy, ecology and behaviour.

Jiang et al. [3] and Castoe et al. [6] sampled five and six non-caenophidian snakes, respectively. Given that these studies found the most dramatic shifts in evolutionary rate and mt genome rearrangement to have occurred along more basal internal branches, it is important to increase sampling of non-caenophidian lineages in order to identify more precisely where/when in snake evolution some key mitogenomic features (including control region duplication) were acquired, and to conduct more stringent tests of the gene- and branch-specific patterns that have been identified. Here we report mt genome sequence data for three additional henophidian (non-caenophidian alethinophidian) snake species: *Anilius scytale, Rhinophis philippinus *and *Charina trivirgata *(see ref [11] for details of the taxonomy of these species). Henophidian sampling is increased further with the inclusion of the tropidophiid *Tropidophis haetianus *and another boid species, *Eunectes notaeus*. In addition to the single scolecophidian included in [3] and two included in [6], two species of *Ramphotyphlops *and one *Typhlops *species are added here. These data are included in new analyses of snake phylogeny and mitogenomic evolution.

## Results

### Mt genomes of *Anilius scytale, Rhinophis philippinus *and *Charina trivirgata*

The entire mt genome of *C. trivirgata*, except for the repeat regions within the control regions, was sequenced. All mt genes in *A. scytale *and *R. philippinus *were sequenced except for the tRNAs flanking the duplicate control region situated within the IQM tRNA cluster (Fig. 1). As previously described for other alethinophidian genomes [1-4,9], the three new mt genomes possess two control regions. This was confirmed in all three species despite incomplete sequencing because it was possible to sequence both the 5' and 3' ends of both control regions in *C. trivirgata *and the 5' ends of both control regions for *A. scytale *and *R. philippinus*. In all three genomes prominent C-rich regions - characteristic of the control region 5' end in the majority of vertebrate mt genomes - were found. We failed to sequence the 3' ends and tRNAs adjacent to CRII for both *A. scytale *and *R. philippinus*, possibly because of extensive repeat regions typically found at the 3' end of the control region. While this paper was in preparation Castoe et al. [10] sequenced the mt genomes of *A. scytale *and *Tropidophis haetianus*. The mt genome of *T. haetianus *also has a duplicate control region and a translocated tRNA^LEU ^gene.

As in other alethinophidian mt genomes, the tRNA^LEU ^(UUR) gene in *C. trivirgata *was found translocated from its typical vertebrate position between the genes 16S and ND1 to downstream of CRII. The same can be assumed for *A. scytale *and *R. philippinus *because this gene was not found between 16S and ND1. As in other alethinophidian snakes, the origin of light strand replication is present in all three newly sequenced mt genomes, with the stem being 12 bp long.

The lengths and GC content of ribosomal and protein-coding genes of the three mt genomes are shown in Table 1. Gene lengths and GC content are similar to those of other snakes. However, COI in *A. scytale *and *R. philippinus *is much shorter than that of *C. trivirgata *(see Table 1). In *Xenopeltis unicolor*, *Python regius*, boids and caenophidians, COI is 1602 sites in length whereas in *T. haetianus*, *Rhinophis philippinus, Cylindrophis ruffus, A. scytale *and scolecophidians COI length is 1536-1581 nucleotides (data not shown). This difference in length is attributable to gaps of variable size occurring at the 3' end of COI (data not shown). The length of ND4 sequences also differs among major snake lineages: 1338 nucleotides for all colubroids except *Achalinus meiguensis *(1353 sites) but 1356 for *Acrochordus granulatus *and henophidians. This difference in length is due to a single gap 130-150 nucleotides into the sequence.

**Table 1:** Gene lengths and GC content of three snake mt genomes

Gene	Length			GC content %		
	
	*A. scytale*	*R. philippinus*	*C. trivirgata*	*A. scytale*	*R. philippinus*	*C. trivirgata*
12S	920	916	927	0.421	0.432	0.457
16S	1492	1479	1481	0.392	0.408	0.431
ATP6	681	680	681	0.374	0.363	0.430
ATP8	168	162	165	0.369	0.364	0.418
CO1	1554	1569	1602	0.411	0.416	0.449
CO2	686	685	687	0.450	0.429	0.453
CO3	784	784	784	0.415	0.427	0.450
CYTB	1117	1116	1117	0.401	0.393	0.440
ND1	967	967	964	0.413	0.414	0.423
ND2	1035	1032	1032	0.389	0.378	0.418
ND3	343	343	343	0.391	0.405	0.461
ND4	1356	1356	1356	0.381	0.381	0.439
ND4L	290	290	291	0.372	0.341	0.395
ND5	1788	1779	1794	0.384	0.389	0.417
ND6	525	522	522	0.337	0.360	0.391

### Phylogenetic analyses

**Figure 2:**
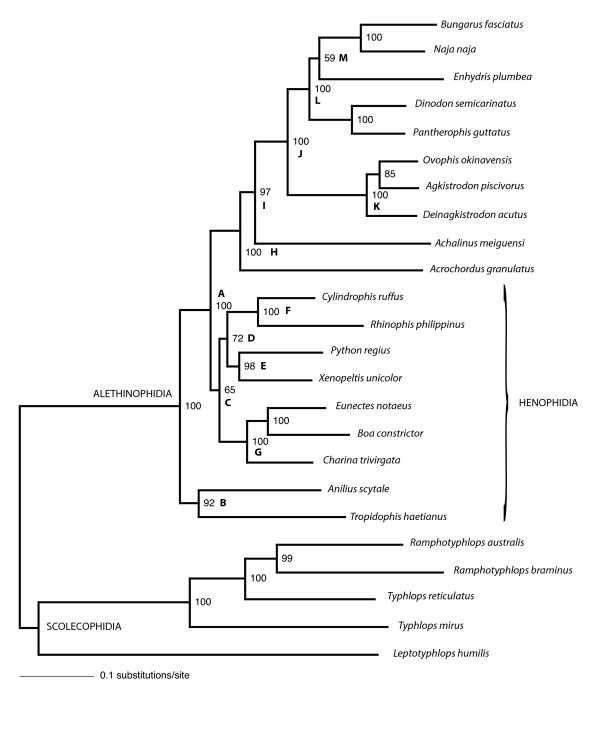
**ML tree based on mt nucleotide data**. ML tree run with data split into four partitions: rRNA, 1st, 2nd and 3rd codon positions, all analyzed under the GTR+I+Γ model. Node support values are expected likelihood weights of local rearrangements (LR-ELW) with 1000 replicates for each node. Higher taxa indicated by labelled nodes are: Boidae (node G), Caenophidia (H), Colubroidea (I), Viperidae (K).

**Figure 3:**
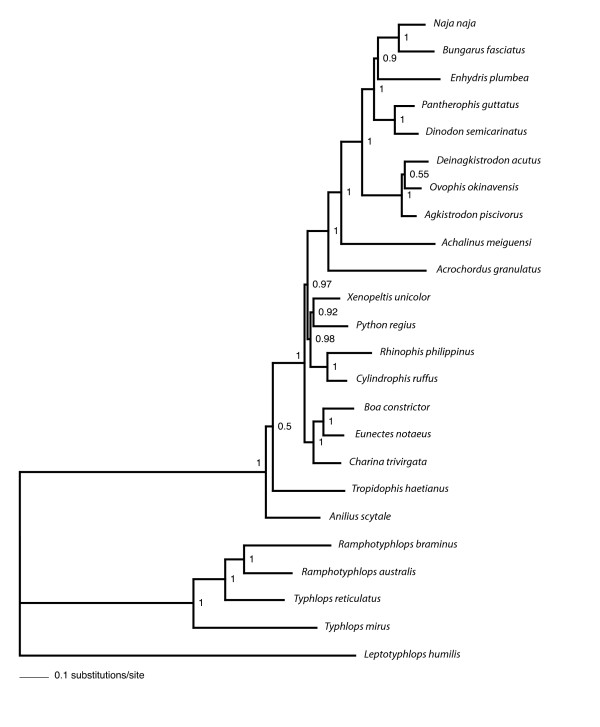
**Bayesian tree based on mt nucleotide data**. Data were run under the CAT-GTR+Γ model in which a free number of categories (partitions) were determined by the PhyloBayes program. Node support values are estimated Bayesian posterior probabilities (BPP).

The total number of nucleotides in the alignment was 12316 (8874 excluding 3rd codon positions), with 3442 amino acid sites. The maximum likelihood (ML) and Bayesian phylogenetic analyses performed in this study yielded trees with unequivocal support for major snake taxa regardless of the method or model used. As we explain below, many of the differences in analytical results were relatively minor and did not conflict strongly in that the contentious nodes were weakly supported. The only strongly conflicting results are shown in Fig. 2 and 3, where boids are sister to either *Python *+ *Xenopeltis + Cylindrophis + Rhinophis *(Fig. 2) or to all alethinophidians except *Anilius *and *Tropidophis *(Fig. 3).

The maximum likelihood (ML) GTR+I+Γ tree based on the 4-partition nucleotide model is shown in Fig. 2. This is identical to a tree recovered by running the 2nd codon positions under the TN+I+Γ model, and the Bayesian analysis in which nucleotide data was split into 26 partitions (see Methods). The Bayes factor for the 26- partition model (H_1_) against the 4-partition model (H_0_) was B_10 _= 0.989 (2lnB_10 _= -0.02), indicating that there was no significant difference between the two models.

Support for nodes labelled A-M (Fig. 2) in all trees is shown in Table 2. The following clades received very high to maximal support in all analyses: all alethinophidians except *Anilius *and *Tropidophis *(referred to here as "core alethinophidians" - node A), *Rhinophis *+ *Cylindrophis *(node F), Boidae (node G), Caenophidia (node H), Colubroids except *Achalinus meiguensis *(node J), Viperidae (node K), 'colubrids' + Elapidae (node L). The clade uniting *Python *+ *Xenopeltis *(node E) received very high support in all trees except the CAT-GTR+Γ tree (see Fig. 3) although support was still fairly strong in this tree (0.92 BPP).

**Table 2:** Results of statistical tree comparisons showing p-values from the six statistical tests performed (see Methods).

Tests	nucleotides	amino acids
ELW	0.315	0.027
BP	0.312	0.026
KH	0.311	0.031
SH	0.311	0.031
WSH	0.311	0.031
AU	0.313	0.026

For both nucleotide and amino acid data, the tree in Fig. 2 was the preferred tree. The p-values shown here reflect the likelihood of topology of the tree in Fig. 3 compared to topology 2. Whereas topology 3 is not rejected with nucleotide data, it is rejected at the 5% level with amino acid data.

Although the inferred trees unanimously supported a clade comprising core alethinophidians (node A), relationships between *Anilius *and *Tropidophis *were not resolved compellingly. *Anilius scytale *+ *T. haetianus *(node B - see Fig. 2) was strongly supported in GTR and mtREV analyses (Table 3) but only moderately supported in the CAT-Poisson+Γ tree. This clade was not recovered at all in the CAT-GTR+Γ tree (Fig. 3), although the alternative - *T. haetianus *and *A. scytale *as successive outgroups to other (core) alethinophidians - was only weakly supported (0.5 BPP). A clade comprising *Rhinophis *+ *Cylindrophis *and *Python *+ *Xenopeltis *(node D) was recovered in all trees and received strong support in Bayesian analyses but only weak to moderate support in ML analyses (Table 3). *Achalinus meiguensis *+ remaining Colubroidea (node I) received very high to maximal support in nucleotide analyses but low support in amino acid analyses with the exception of the mtREV+I+Γ analysis run with the program PhyloBayes (Table 3). In the CAT-Poisson+Γ tree *A. meiguensis *joins with *Acrochordus granulatus *with weak support (data not shown). *Enhydris plumbea *is the sister to Elapidae (node M) in nucleotide analyses and the PhyloBayes mtREV+I+Γ analysis (Table 3) but this clade received moderately high support only in the MrBayes nucleotide analysis. Most amino acid analyses recovered *E. plumbea *to be the sister of a clade comprising colubrines and elapids (see Additional file 1), but this never received strong support.

**Table 3:** Support values from the various analyses for nodes A-M as indicated in Fig. 2

		A	B	C	D	E	F	G	H	I	J	K	L	M
nt	ML	100	92	65	72	98	100	100	100	98	100	100	100	59
	MLTN	100	93	66	71	98	100	100	100	98	100	100	100	57
	MLB	100	95	64	66	99	100	100	100	97	100	100	100	63
	MB	1	1	1	1	1	1	1	1	1	1	1	1	1
	PB	1	-	-	0.98	0.92	1	1	1	1	1	1	1	0.9

aa	ML	100	99	96	66	99	100	100	100	51	100	100	100	-
	MLB	100	100	98	58	99	100	100	100	55	100	100	100	-
	MB	1	1	1	1	1	1	1	1	0.54	1	1	1	-
	PBC	1	0.73	-	0.99	0.99	1	0.99	1	-	1	1	1	-
	PBM	1	1	-	1	1	1	1	1	0.95	1	1	1	0.84

The values given are for expected likelihood weights of local rearrangements (LR-ELW), bootsrap, and Bayesian posterior probabilities (BPP). ML: maximum likelihood LR-ELW; MLTN: ML LR-ELW for analyses where 2nd codon positions were analyzed under the TN+I+Γ model; MLB: ML bootstrap; MB: BPP for analyses performed with MrBayes; PB: BPP for analyses performed with PhyloBayes (CAT-GTR for nucleotides); PBC: BPP for PB analyses using CAT-Poisson model; PBM: BPP for PB analyses using the mtREV+I+Γ model. nt: nucleotide analyses; aa: amino acid analyses.

Only node C (Fig. 2) is strongly supported or conflicted in different analyses. MrBayes analyses and amino acid ML analyses recovered node C with very high or maximal support (Fig. 2, Table 3), whereas this node received <70% bootstrap/LR-ELW support in ML nucleotide analyses. CAT-GTR and CAT-Poisson analyses recovered the alternative topology shown in Fig. 3 with ≥ 95 BPP. Interestingly, the mtREV analysis run with PhyloBayes also recovered the boid relationship shown in Fig. 3, albeit with only 0.65 BPP (see Additional file 2). Statistical tree comparisons with nucleotide data could not reject the tree shown in Fig. 3 as significantly suboptimal compared to that in Fig. 2 (Table 2), the difference in likelihood between the two trees only negligible (ΔlnL = 4.8). With amino acid data however, the CAT topology (ΔlnL = 23.47; Fig. 3) was rejected by all statistical tests at the 0.05 significance level (Table 2).

### Relative rates of molecular evolution of mt genes

**Figure 4:**
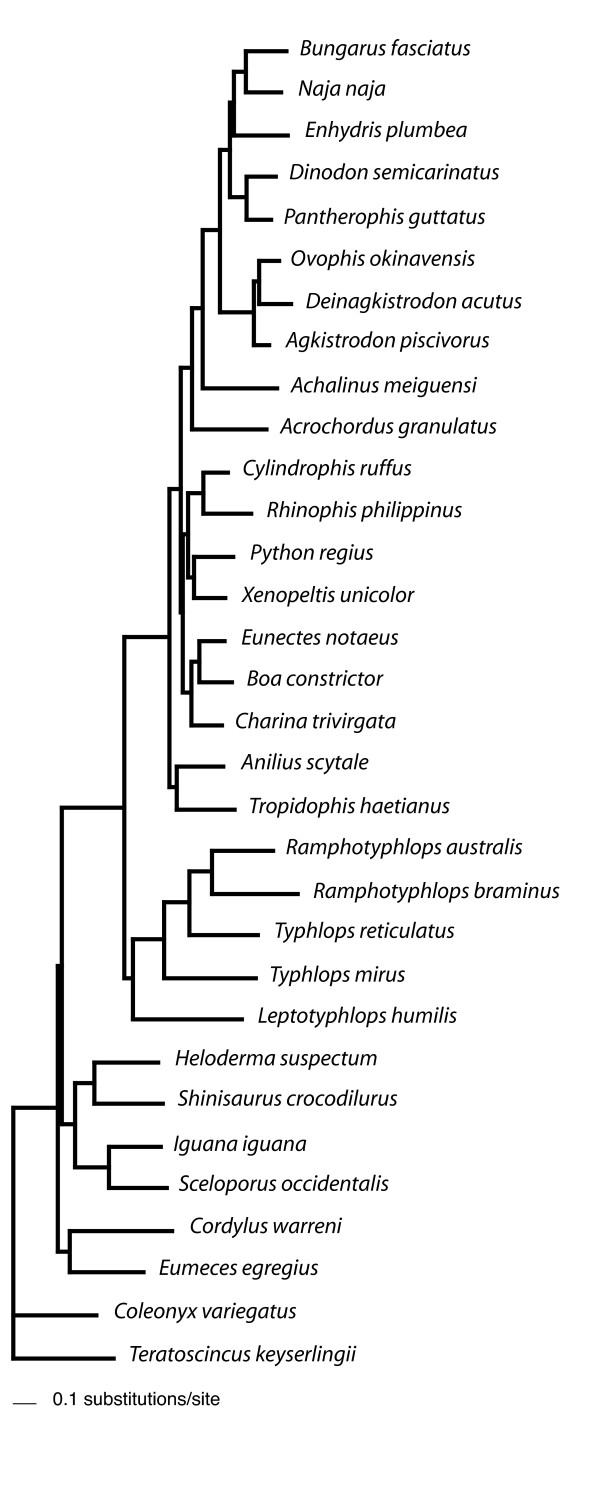
**ML phylogram showing branch lengths for the topology in Fig. 2 estimated with rRNA nucleotide data**. Analysis was run under the GTR+I+Γ model. Note that branch lengths are drawn to the same scale in Figs. 4, 5 and 6 to aid comparison.

**Figure 5:**
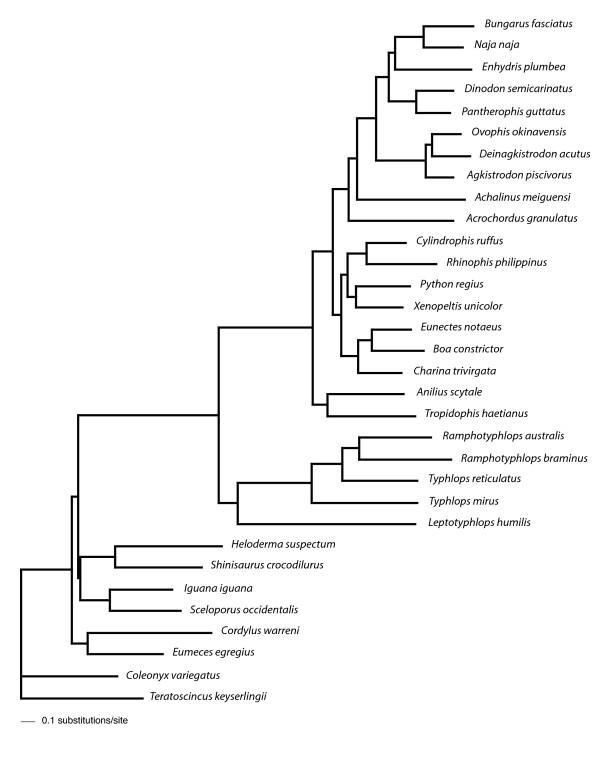
**ML phylogram showing branch lengths for the topology in Fig. 2 estimated with protein-coding gene nucleotide data**. Branch lengths estimated under the GTR+I+Γ model and with 1st, 2nd and 3rd codon positions weighted 2, 1 and 5 respectively.

**Figure 6:**
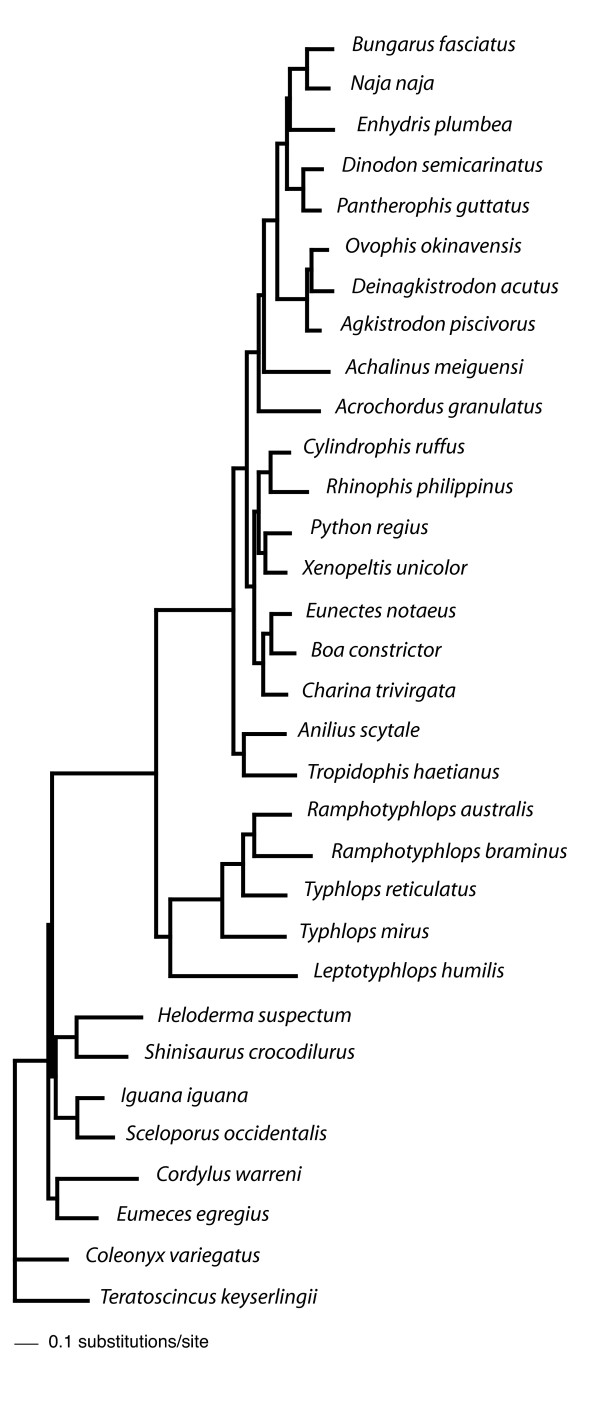
**ML phylogram showing branch lengths for the topology in Fig. 2 estimated for amino acid data**. Branch lengths in this analysis were estimated under the mtREV+I+Γ model.

Relative nucleotide branch lengths of rRNA genes and protein-coding genes can be visualized in Figs. 4, 5 and 6. As expected, rRNA branches (Fig. 4) are substantially shorter overall than protein-coding branches (Fig. 5). CAT analyses also produced the same trend (data not shown). Branches leading to Serpentes, Alethinophidia, Typhlopidae and *L. humilis *show especially accelerated amino acid change relative to rRNA nucleotide branch lengths (Fig. 6). Our phylogenetic analyses produced two alternative resolutions of basal nodes among core henophidians (Figs. 2 and 3) and so the rates analyses were carried out twice. The different trees barely affected relative evolutionary rates, and the results shown here are based on the phylogeny in Fig. 2.

**Figure 7:**
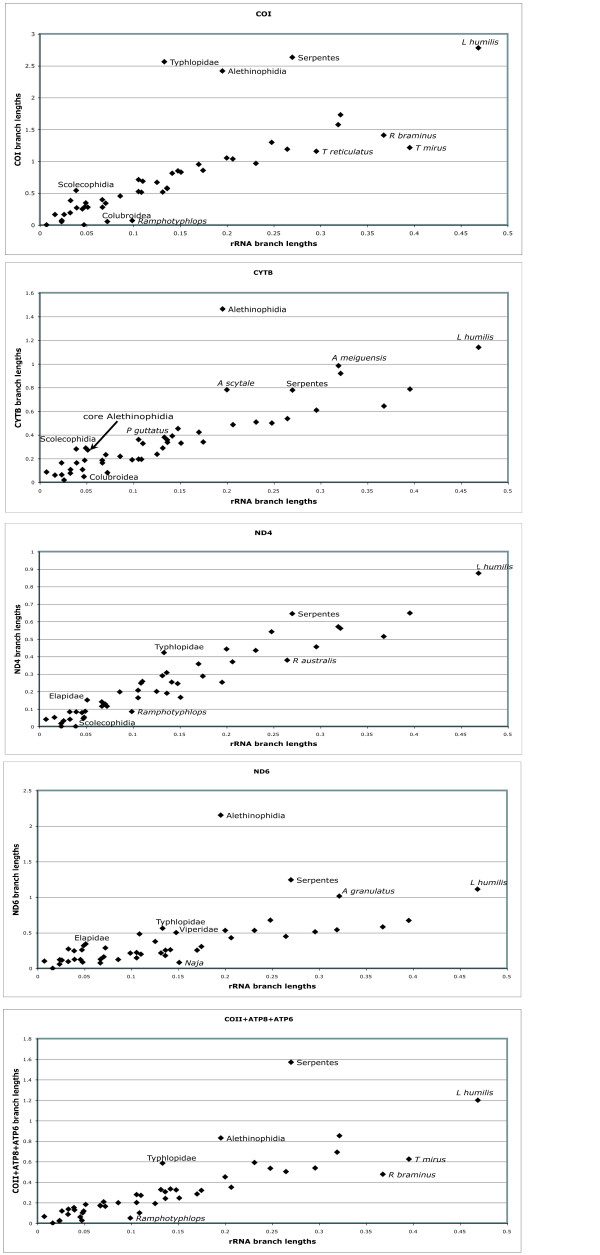
**Plots showing rRNA nucleotide branch lengths vs. individual protein-coding gene nucleotide branch lengths**. The plots shown here are for the following protein-coding genes: COI, CYTB, ND4, ND6, COII+ATP8+ATP6. Branch lengths were estimated using ML under the GTR+I+Γ model and 1st, 2nd and 3rd codons weighted 2, 1 and 5, respectively.

**Figure 8:**
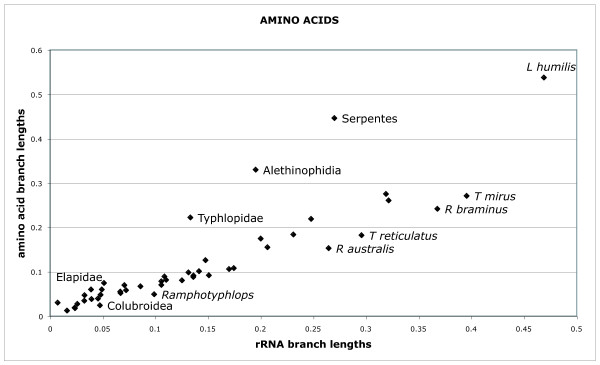
**Plot showing rRNA nucleotide branch lengths vs. amino acid branch lengths**. The branch lengths used for this plot were taken from Fig. 6, where branch lengths were estimated under the mtREV+I+Γ model using concatenated amino acid data.

Patterns of evolution for different genes along different branches found in this study are broadly concordant with those presented by Jiang et al. [3]. Branch lengths of rRNA and protein-coding genes are generally positively correlated, positive deviances from this correlation being most prominent for CO genes, ATP genes, CYTB and ND6 (Fig. 7), with only slight deviances in other genes/clusters. These same trends were evident under both of the nucleotide partitioning schemes used (see Methods section 2.6), and in the plot of rRNA vs. amino acid branch lengths (Fig. 8), the latter suggests that markedly raised protein-coding gene evolutionary rates are not simply due to large numbers of synonymous changes. The most dramatic acceleration in relative rate of evolution occurred along the branches leading to Serpentes and to Alethinophidia, for which there was an acceleration in most protein-coding genes. Relative rate accelerations were found along the branch leading to *A. scytale + T.haetianus *in COI and ND6 (Fig. 7). There were no dramatic relative rate shifts in overall protein-coding gene evolution along the branch leading to core alethinophidians, although there was a slight relative acceleration in CYTB evolution here (Fig. 8). The other branch that showed the most dramatic elevation in protein-coding gene relative rates was that leading to Typhlopidae, which had elevated relative rates in COI, CO2, ATPs, ND2, ND4, ND5 and ND6. However, relative rates of evolution in most protein-coding genes along terminal typhlopid branches and the internal branch leading to *Ramphotyphlops *are moderately decelerated, also reflected in the amino acid plot (Figs. 7 and 8). In the rRNA tree (Fig. 4), typhlopids have the longest branches of all snake taxa except *A. granulatus *and *L. humilis*, the latter having elevated rates in all genes. The branch leading to Scolecophidia has relatively accelerated rates for COI, CO3, CYTB, ND3, ND4L and ND6, but dramatically decelerated for ND2 and ND4. The branch leading to Colubroidea occurs below the scatter distribution in the graphs for most genes, ND6 being a notable exception (Fig. 7). This deceleration is also seen in the amino acid plot (Fig. 8). The branch leading to Elapidae shows slight accelerations in rate in genes ND1, ND4 and ND6, which is also reflected in the amino acid plot (Fig. 8). Rates of evolution in ND1 show indications of relative acceleration in several terminal branches within Henophidia.

## Discussion

### Relationships among the major lineages of snakes

The phylogenetic relationships among non-caenophidian snakes has fundamental importance for the interpretation of early snake evolution [7,12,13], but despite increased geno- and phenotypic character sampling over recent years, explicit quantitative phylogenetics has yet to deliver an entirely robust tree. Our analyses confirmed strong support for many nodes that have been robustly resolved in other studies, for example Colubroidea, Caenophidia, Colubridae, and nodes that were supported by other molecular studies but found to be equivocal or generally not recovered by morphological studies (*Python*+*Xenopeltis*, core alethinophidians, Cylindrophiidae + Uropeltidae). However, as with previous molecular studies, most deeper henophidian nodes could not be resolved robustly in this study. Low node support is often associated with short internal branches that can result from incongruence within data and/or too few character changes (e.g. [14,15]). As is evident in Figs. 2 and 3, there are many short internal branches among henophidians, but that this is not a feature of mitochondrial data alone is indicated by relatively short branches and problematic nodes in analyses using nuclear data also [15].

Prior to the first broad-scale molecular snake phylogenies, morphological phylogenies had reached a consensus on several relationships among major snake lineages [7]. Morphologists were not entirely surprised by some of the new molecular findings because they mostly affected the less robustly inferred regions of morphological phylogenies, including the possible close relationship between pythons, *Xenopeltis*, and *Loxocemus*, the non-monophyly of macrostomatan alethinophidians and the non-monophyly of dwarf boas [16]. However, the sister-group relationship between *Anilius *and tropidophiids proposed by several molecular studies [7,17-20] remains a notable sticking point between molecular and morphological studies [7], especially considering that recovery of this clade in larger nuclear gene studies [15] negates dismissal of this hypothesis as an aberration of mt data alone. It might be noted that this relationship was not very strongly supported in our analyses.

### Mitogenomic evolution in snakes

Previous studies (e.g. refs [3,4]) have mapped the duplication of the control region and translocation of tRNA^LEU ^to the internal branch leading to extant alethinophidians, but incomplete taxon sampling (especially the absence of *Anilius*) had left this proposition incompletely tested. Our identification of these same derived mitogenomic features in *A. scytale *provides empirical support for this. The same features are present in all alethinophidian genomes sequenced to date.

Our analyses of relative rate variation in mt gene evolution across the snake tree produced several results in concordance with the study of Jiang et al. [3], including overall dramatically accelerated (relative to rRNAs) protein-coding gene evolutionary rates along the internal branches leading to Serpentes and to Alethinophidia though with notable gene- and branch-specific variation (e.g., dramatic relative acceleration in evolution of COI, COII, ATP8, ATP6, ND6 and CYTB along branch leading to Alethinophidia). Notable differences between our results and those of Jiang et al. [3] are: no snake branches disproportionately longer for rRNA (Fig. 4) than combined protein-coding (Fig. 5) genes that would suggest dramatic relatively accelerated rRNA evolution; no notable acceleration in relative rate of CYTB evolution (and less dramatic increase in ND2) along branch leading to Serpentes; internal branch leading to Colubroidea with relatively decelerated COI and CYTB evolution (Fig. 7). The latter is potentially dependent on the position of *Achalinus meiguensis*, which was not resolved robustly here, but analyses with this taxon excluded produced the same patterns (data not shown).

This study has generated new findings, notably: an overall dramatically accelerated protein-coding gene evolution on the branch leading to Typhlopidae; terminal branches within Typhlopidae and the internal branch leading to *Ramphotyphlops *generally falling below the main distribution in gene-gene plots (Fig. 7); relative acceleration in COI and ND6 along the branch leading to *A. scytale + T. haetianus*; the terminal branch leading to *A. scytale *slightly accelerated for CYTB, ND4, ND5 and ND6; further relative acceleration in CYTB and ND6 evolution along branch leading to core alethinophidians; relative acceleration in ND1, ND4 and ND6 along the branch leading to Elapidae. Although we did not carry out the in-depth analytical tests of positive selection or protein structural modelling performed by Castoe et al. [6], the rRNA vs. protein-coding amino acid branch length plot (Fig. 8) demonstrates that some of the acceleration in molecular evolution along the branches leading to Serpentes, Alethinophidia and Typhlopidae is likely to be adaptive in that it leads to changes in protein sequences, and is not simply an increase in 'silent' substitutions.

Our greater taxon sampling enabled us to identify additional trends in mt gene evolution for some snake clades. Within Scolecophidia, we found dramatic relative acceleration in protein-coding gene evolution along the branch leading to Typhlopidae but subsequent relative deceleration (relative acceleration in rRNA evolution) along all terminal typhlopid branches. The terminal branch joining *Leptotyphlops humilis *to the rest of the tree appears above the main distribution in most gene-gene plots (for nucleotide and amino acid data), but comparison with other lineages is complicated because only one leptotyphlopid mt genome has been sequenced. Additional sampling of leptotyphlopids is required to clarify mitogenomic evolution along this branch. The inclusion of *L. humilis *is not responsible for the rapid protein evolution identified along the branch leading to Typhlopidae because this branch is similarly long in amino acid trees that exclude *L. humilis *(data not shown). The generally long branches within Scolecophidia and seemingly strongly fluctuating rate dynamics suggest that mitogenomic evolution within this group is worthy of greater attention. Although scolecophidians represent one half of the basal divergence among living snakes they are often overlooked, perhaps because of their superficial morphological and ecological homogeneity, which is, however, deceptive [21].

Castoe et al. [6] observed that dramatic adaptive shifts in the evolution of metabolic proteins occurred along the branch leading to Alethinophidia and so were potentially temporally associated with the origin of a duplicated control region, and they suggested that these shifts along this internal branch were causally associated with major anatomical, ecological, and behavioural changes such as an ecological niche shift to a non-burrowing lifestyle, increased body size, increased skull kinesis and gape and prey size, development of specialized venom proteins, and the ability of individuals to dramatically remodel their organs and physiology. The scenario of a single major switch from a burrowing to non-burrowing (and macrostomatan) condition early in alethinophidian history has been eroded by some of the more recent phylogenetic results for henophidian snakes, so that identifying broad suites of phenotypic adaptive change along the branch leading to Alethinophidia is not as trivial as previously assumed. However, the rapid remodelling of metabolic proteins before the burrowing *Anilius *diverged from other alethinophidians and, importantly, evidence of elevated relative rates of metabolic protein evolution in the branch leading to the exclusively burrowing Scolecophidia (which lack a duplicated control region) rejects the exact correlations that underpin some of the precise aspects of the hypotheses previously proposed to explain the observed mitogenomic evolutionary patterns.

## Conclusions

Phylogenetic analyses of complete mt genome data strongly support many of the clades identified in previous studies such as core Alethinophidia, Colubroidea and Cylindrophiidae + Uropeltidae, but deeper henophidian nodes were not resolved with compelling support. Extended taxon sampling allowed us to identify dramatic acceleration of metabolic protein evolution within Scolecophidia in addition to the rate shifts identified by Jiang et al. [3]. In addition, control region duplication and most of the rate acceleration in mt genes occurred before *Anilius *and *Tropidophis *branched off from the rest of Alethinophidia, although less dramatic acceleration in protein-coding mt genes occur in these lineages. Our new data discount a consistent link between extraordinary bursts of mitogenomic evolution early in snake history and control region duplication or transition to a non-burrowing lifestyle and macrostomatan feeding ecology. Further taxon sampling for mt genomes can contribute to an improved phylogenetic understanding and will be crucial for further investigation of mitogenomic evolution. We identify priority taxa for both phylogenetic and mitogenomic studies to be *Anomochilus*, bolyeriids, the monotypic *Xenophidion *and *Loxocemus*, and more scolecophidians (especially at least one anomalepidid and more leptotyphlopids).

## Methods

### Taxon sampling, DNA extraction, PCR and sequencing

We analysed complete/near-complete mt sequence data for 24 species of snakes (22 genera), currently available through Genbank and those sequenced here (Table 4). Thus, we include approximately double the number of species analysed by Jiang et al. [3], with 8 of 13 of the additional species being non-caenophidians and therefore potentially clarifying the nature of the mitogenomic evolution occurring along more basal internal branches in the snake tree. We generated new sequence data for single specimens of *Anilius scytale *(Kaw Mountains, French Guiana: no voucher), *Rhinophis philippinus *(National Museum, Colombo, Sri Lanka field number MW 1740), and *Charina trivirgata saslowi *(sourced through the pet trade: no voucher). Total DNA was extracted from muscle tissue using the organic extraction method. Overlapping fragments of mtDNA were amplified with Ex-Taq and Z-Taq (Takara) polymerases and using conserved primers designed in this and previous studies [9,22]. PCR-products were sequenced using the ABI automated sequencing system. The taxonomy we use is shown in Table 4 and Fig. 2.

**Table 4:** Snake species sampled in this study and their mt genome Genbank accession numbers.

Scolecophidia	Leptotyphlopidae	*Leptotyphlops humilis*	NC 005961
	Typhlopidae	*Ramphotyphlops australis*	AM 236346
	Typhlopidae	*Ramphotyphlops braminus*	NC 010196
	Typhlopidae	*Typhlops reticulatus*	NC 010971
	Typhlopidae	*Typhlops mirus*	AM 236345
Alethinophidia: "Henophidia"	Aniliidae	Anilius scytale*	GQ 200593
	Tropidophiidae	*Tropidophis haetianus*	NC 012573
	Uropeltidae	Rhinophis philippinus*	GQ 200594
	Cylindrophiidae	*Cylindrophis ruffus*	NC 007401
	Xenopeltidae	*Xenopeltis unicolor*	NC 007402
	Pythonidae	*Python regius*	NC 007399
	Boidae	Charina trivirgata*	GQ 200595
	Boidae	*Eunectes notaeus*	AM 236347
	Boidae	*Boa constrictor*	AM 236348
Alethinophidia: Caenophidia	Acrochordidae	*Acrochordus granulatus*	NC 007400
Alethinophidia: Caenophidia: Colubroidea	Viperidae	*Ovophis okinavensis*	NC 007397
	Viperidae	*Agkistrodon piscivorus*	NC 009768
	Viperidae	*Deinagkistrodon acutus*	NC 010223
	Homalopsinae/dae	*Enhydris plumbea*	NC 010200
	Incertae sedis	*Achalinus meiguensis*	NC 011576
	Elapidae	*Naja naja*	NC 010225
		*Bungarus fasciatus*	NC 011393
	Colubrinae	*Dinodon semicarinatus*	NC 001945
	Colubrinae	*Pantherophis guttatus*	AM 236349

Asterisks indicate taxa newly sequenced for this study.

### Data assembly and alignment

Sequences were edited and assembled using EditView 1.0.1 (Perkin-Elmer). Gene organization within the mtDNA sequences was inferred by comparisons with other snake genomes using the program SeAl (v2.0a11). The twelve H-strand encoded mt gene sequences from all taxa were aligned using MAFFT [23] and concatenated. 12S and 16S ribosomal RNA genes were aligned using the program T-COFFEE [24] incorporating information from secondary structure [25]. All mt gene alignments were subsequently inspected manually. Alignment-ambiguous characters (where the program was equivocal in the placing of certain gaps) were excluded from the dataset.

### Data partitioning and modelling

The data were analysed separately as nucleotide and amino acid sequences. The nucleotide data were subdivided into three partitions (rRNAs; protein-coding 1st codon positions; 2nd codon positions). The phylogenetic signal in 3rd codon positions was investigated by running preliminary parsimony and neighbour joining analyses using PAUP* [26], and found to contain substantially more signal when analysed under RY coding than as raw nucleotide data. In RY coding, pyrimidine bases C and T are analysed as one character, as are purine bases A and G. RY-coded third positions were thus included as a fourth partition in ML and MB analyses. Third codon positions were not included in PhyloBayes analyses because RY coding is not possible on this platform (N. Lartillot, pers. comm.).

Whether further partitioning of the data would yield an improvement over the 4-partition dataset was investigated by calculating the Bayes Factor (B_10_), which is robust against Type I error ([27], J. Brown, pers. comm.). A dataset with 26 partitions (12S rRNA, 16S rRNA, ND1, ND2, CO1, CO2+ATP8+ATP6, CO3+ND3+ND4L, ND4, ND5, CYTB, with all protein-coding genes being split into three partitions comprising 1st, 2nd and 3rd codon positions) represented H_1 _and the 4-partition dataset represented H_0_. Third codon positions were coded as RY. Both datasets were analysed using the program MrBayes (see below). The harmonic means were calculated using the program Tracer (v 1.4) and incorporated into the formula: B_10 _= harmonic mean L_1_/harmonic mean L_0_. The Bayes Factor was interpreted as in Kass & Raftery [28].

The model of choice for each partition was determined using the program Modeltest [29]. Both the Likelihood Ratio Test (LRT) and Akaike Information Criterion (AIC) found the GTR+I+Γ model [30,31] as best fitting for all partitions except 2nd codon positions, for which LRT found TN+I+Γ [32] as best fitting.

Amino acid sequences were analyzed using the mtREV+I+Γ model [33]. The categories model with GTR (CAT-GTR) and CAT-Poisson models [34,35] were also used to analyse nucleotide and amino acid data, respectively.

### Phylogenetic analyses

Maximum likelihood (ML) analyses were performed in Treefinder [36]. For nucleotide sequences each analysis was started from ten different initial trees to increase the amount of tree space explored. Amino acid analyses were initiated from five different start trees. Support for nodes was determined using expected likelihood weights of local rearrangements (LR-ELW) with 1000 replicates for each node. Both nucleotide and amino acid data were also bootstrapped with 200 and 100 replicates, respectively. Bayesian analyses run under GTR+I+Γ and mtREV+I+Γ models were performed using MrBayes [37,38] freely available through Bioportal cluster [39]. Nucleotide and amino acid data were run for 10,000,000 and 5,000,000 generations, respectively. Convergence was checked using Tracer. Analyses using CAT models were run with the PhyloBayes 2.3 program package [34] also available on Bioportal. Here amino acid data were analyzed using both CAT-Poisson and mtREV+I+Γ models. Convergence was checked with the *bpcomp *program, whereby convergence was reached if the maxdiff value = <0.1.

Because our primary concern was the relationships of snake lineages, the main phylogenetic analyses were not conducted with (distant) non-snake outgroups. Instead, we rooted Scolecophidia with Alethinophidia and vice versa, based on this being a well-supported relationship in previous morphological and molecular estimates of snake phylogeny [3,7,13,15,40].

### Tree comparisons

Alternative topologies were compared statistically using the program Treefinder. Both nucleotide and amino acid data were analyzed. Six statistical tests were carried out simultaneously: Bootstrap Probability [41], Expected-Likelihood Weights (ELW) [42], Kishino-Hasegawa (KH) [43], Shimodaira-Hasegawa (SH), Weighted SH (WSH) [44] and Approximately Unbiased (AU) [45], all implemented in Treefinder [36].

### Relative rates of evolution of mt genes

In accordance with Jiang et al. [3], we analysed temporal patterns of molecular evolution, measured by nucleotide branch length, among the different branches of the snake tree for individual protein-coding genes relative to branch lengths for 12S + 16S rRNAs. We also carried out similar analyses based on amino acid sequences in order to gain some insight as to whether notably rapid nucleotide change in protein-coding genes was associated with (potentially adaptive) change at the protein level. Because we wanted to include estimates of the length of the branch connecting snakes to other squamates, we included the following selection of lizard outgroups (Genbank accessions in parentheses): *Gekko gecko *(NC 007627), *Coleonyx variegatus *(NC 008774), *Cordylus warreni *(NC 005962), *Eumeces egregius *(NC 000888), *Iguana iguana *(NC 002793), *Sceloporus occidentalis *(NC 005960), *Heloderma suspectum *(NC 008776), *Shinisaurus crocodilurus *(NC 005959). The phylogeny used to calculate branch lengths was fixed, with the relationships among snakes based on our phylogenetic results, and those among lizards following refs [46] and [47].

The branch lengths of rRNA and protein-coding genes were compared by firstly estimating branch lengths separately for rRNAs and all protein-coding genes using both nucleotide and amino acid data. Because of the erosion of signal at 3rd codon positions (see section 2.3) 1st, 2nd and 3rd codon positions were weighted 2, 1 and 5, respectively. ND6 was analysed as a separate partition because of its aberrant base composition. The amino acid analysis was run without partitioning. Secondly, rRNA branch lengths were compared with those for individual protein-coding genes in bivariate plots. Following Jiang et al. [3] the short genes CO2, ATP8 and ATP6 were concatenated into one partition to reduce stochastic error, as were genes CO3, ND3 and ND4L. Two different partitioning schemes were used for each protein-coding gene/cluster: a) 1-partition with 1st, 2nd and 3rd codon positions assigned different weights as described above and b) equal-weighted 3-partitions based on codon position, with RY coding of 3rd codon positions. This second partitioning scheme was used as an alternative to amino acid data that, because of the shorter gene lengths, would increase the risk of stochastic error. The use of RY coding reduces variation at 3rd codon positions considerably, which means that the branch lengths would more accurately reflect evolution at 1st and 2nd codon positions. Nucleotide and amino acid branch lengths were estimated in Treefinder. Branch lengths for each protein-coding gene/cluster were plotted against the respective branch lengths for rRNAs in Microsoft Excel.

## Authors' contributions

DD partly conceived the project, generated new sequence data, performed the analyses and co-wrote the manuscript. DJG partly conceived and designed the project, collected material in the field and co-wrote the manuscript. All authors read and approved the final manuscript.

## Acknowledgements

Assistance was provided to DJG during fieldwork to collect samples used in this study by O Ballou, J-A Cerda, P Gaucher, A Kupfer, and M Wilkinson (*Anilius scytale*) and JL Gower, GLK Kariyawasam, H Lokugamage, Y Mapatuna, F Naggs, I & W Perera, D Raheem, SRMS Samaradiwakara, M Wilkinson, and KASR Wickramanaike (*Rhinophis philippinus*). The Director of the Department of National Museums, Colombo, Sri Lanka is thanked for granting loan of material. We thank *Tropikhuset *in Malmö for supplying the *C. trivirgata *specimen, and PG Foster and B Hallström for help with running some of the analyses. R Crozier and J Brown on their input in running Bayesian analyses and Bayes Factor analysis. D San Mauro provided constructive criticism of an earlier draft. This work was supported by the Jörgen Lindström stipendium, Nilsson-Ehle (Kungliga Fysiografiska Sällskapet I Lund) Fund, Leverhulme Trust Grant F/00696/F and Darwin Initiative Grant 162/08/214.

## Supplementary Material

Additional file 1 **The ML tree based on amino acid data under the mtREV+I+Γ model**. The support values are LR-ELW. In this tree, and in most amino acid phylogenetic analyses, *Enhydris plumbea *is the sister-group of elapids and colubrids, in contrast to nucleotide analyses, in which *E. plumbea *is the sister-group of elapids. Click here for file

Additional file 2 **The Bayesian MTR+I+Γ tree produced using the program PhyloBayes**. The support values are BPP. This analysis gives weak support to the CAT topology (Fig. 3), i.e. boids sister-group to all alethinophidians with the exception of *Anilius *and *Tropidophis*, in conflict with node C (Fig. 2). This is in contrast to other MTR+I+Γ analyses that give strong support to node C. Click here for file
